# Functional Implications of Novel Human Acid Sphingomyelinase Splice Variants

**DOI:** 10.1371/journal.pone.0035467

**Published:** 2012-04-27

**Authors:** Cosima Rhein, Philipp Tripal, Angela Seebahn, Alice Konrad, Marcel Kramer, Christine Nagel, Jonas Kemper, Jens Bode, Christiane Mühle, Erich Gulbins, Martin Reichel, Cord-Michael Becker, Johannes Kornhuber

**Affiliations:** 1 Department of Psychiatry and Psychotherapy, University Hospital, Friedrich-Alexander-University Erlangen-Nuremberg, Erlangen, Germany; 2 Institute of Biochemistry, Emil-Fischer-Centre, Friedrich-Alexander-University Erlangen-Nuremberg, Erlangen, Germany; 3 Department of Molecular Biology, University of Duisburg-Essen, Essen, Germany; 4 Leibniz Institute for Age Research – Fritz Lipmann Institute and Center for Sepsis Control and Care at Jena University Hospital, Jena, Germany; University of Kansas Medical Center, United States of America

## Abstract

**Background:**

Acid sphingomyelinase (ASM) hydrolyses sphingomyelin and generates the lipid messenger ceramide, which mediates a variety of stress-related cellular processes. The pathological effects of dysregulated ASM activity are evident in several human diseases and indicate an important functional role for ASM regulation. We investigated alternative splicing as a possible mechanism for regulating cellular ASM activity.

**Methodology/Principal Findings:**

We identified three novel ASM splice variants in human cells, termed ASM-5, -6 and -7, which lack portions of the catalytic- and/or carboxy-terminal domains in comparison to full-length ASM-1. Differential expression patterns in primary blood cells indicated that ASM splicing might be subject to regulatory processes. The newly identified ASM splice variants were catalytically inactive in biochemical *in vitro* assays, but they decreased the relative cellular ceramide content in overexpression studies and exerted a dominant-negative effect on ASM activity in physiological cell models.

**Conclusions/Significance:**

These findings indicate that alternative splicing of ASM is of functional significance for the cellular stress response, possibly representing a mechanism for maintaining constant levels of cellular ASM enzyme activity.

## Introduction

Acid sphingomyelinase (ASM, EC 3.1.4.12) is a glycoprotein localised primarily to the lysosome where it catalyses the breakdown of sphingomyelin to ceramide and phosphorylcholine at a pH optimum of 5 [1]. On the one hand, ASM activity changes the lipid composition of membranes. On the other, ASM generates the bioactive lipid ceramide. According to the ‘rheostat concept’ [2], increased ceramide levels promote apoptosis, whereas an increase in the phosphorylated metabolite of ceramide, sphingosine-1-phosphate, counteracts this effect by inducing proliferation [3]. The dynamic balance between these bioactive lipids is influenced by the activities of acid ceramidase, sphingosine kinase, sphingosine-1-phosphate phosphatase, sphingosine-acetyltransferase and ASM. Among these enzymes, ASM holds a prominent position because it generates the first bioactive molecule in the rheostat. Consequently, abnormal ASM activity is linked to different pathological conditions. Dramatically decreased ASM activity, due to inherited sequence variations in the *SMPD1* gene coding for ASM, is the cause of the type A and B forms of Niemann-Pick disease [4]. Elevated ASM activity is associated with different neuro-psychiatric disorders like Alzheimer’s dementia [5], status epilepticus [6], alcoholism [7] and major depression disorder [8]. Due to these pathological consequences, the tight regulation of ASM activity is crucial for normal cellular function.

A variety of external stress stimuli leads to the activation of ASM, such as CD95 ligand [9], lipopolysaccharide [10], ionising radiation [11], cisplatin [12] and tumour necrosis factor-α [13] to name a few. Different stimuli lead to an upregulation of ASM at the transcriptional level [14], [15], [16], [17], [18], representing a slow regulatory response. In contrast, post-translational modifications act as a fast regulatory response to external stimuli. *In vitro* activation of ASM results from interaction with zinc [19] and copper ions. The latter mediates ASM dimerisation via a cysteine (at position p.631) at the carboxy (C)-terminus of the protein [20]. An activating effect is also exerted by protein kinase δ, which phosphorylates a serine residue (at position p.510) located in the C-terminal domain of ASM [21].

Four different ASM isoforms have been identified to date, although only one has been reported to be catalytically active. Consequently, ASM regulation may also involve alternative splicing. Thus far, only the full-length transcript (GenBank Accession Number NM_000543.4; referred to as ASM-1 in this manuscript) has been shown to code for an active enzyme. This transcript is composed of exons 1 to 6 with a total coding length of 1896 bp. Relative to ASM-1, the alternatively spliced transcript ASM type 2 (NM_ 001007593.1; termed ASM-2) contains a 40 bp insertion derived from intron 2 and lacks exon 3. The alternatively spliced transcript ASM type 3 (NR_027400; termed ASM-3) lacks exon 3. This creates a frameshift in the coding sequence and leads to premature termination during translation [22], [23]. An additional ASM transcript, which lacks 652 bp of exon 2, was isolated from human brain tissue (AY649987.1; referred to as ASM-4).

In this study, we identified three hitherto unknown alternatively spliced human ASM transcripts, which we termed ASM-5, ASM-6 and ASM-7. The characterisation of intrinsic biochemical properties indicated that none of the newly identified ASM variants is catalytically active *in vitro*. However, the new variants exert a dominant-negative effect on cellular ceramide content and on ASM activity. This study provides the first evidence of a regulatory effect of alternatively spliced ASM transcripts.

## Results

### Identification of New ASM Splice Variants in Human Cells

**Figure 1:**
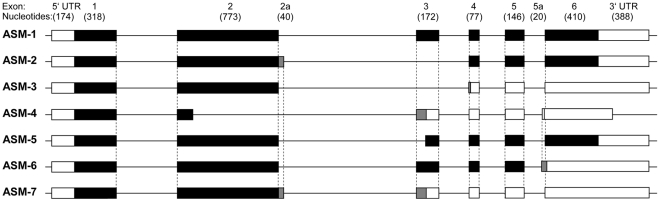
A schematic of the genomic alignment of full-length ASM-1 and all known splice variants. ASM-1 constitutes the reference sequence to which all other ASM variants are compared. The novel variant ASM-5 lacks part of exon 3, resulting in a shorter (1818 bp) coding sequence. Novel variants ASM-6 and ASM-7 contain different intronic insertions. Consequently, their transcript lengths are 1907 bp and 1921 bp, respectively. The insertions create frameshifts in the coding sequence and introduce premature stop codons, which terminate translation after 1521 bp and 1197 bp, respectively. For information on the previously described variants ASM-2, -3, -4 see [22], [23]. In this schematic, lines represent genomic sequences, black boxes indicate exons translated as in ASM-1, grey boxes refer to exonic sequences translated into different amino acids due to a frameshift or coding sequences that correspond to introns in ASM-1, and white boxes denote exonic sequences that follow a premature termination codon.

Because a variety of orthologous transcripts has been described for ASM in public databases, we investigated the human ASM transcriptome more closely. In a first screen on human neuroglioma cells and PBMCs, 24% of the 17 analysed ASM transcripts had undergone alternative splicing. We identified three ASM transcripts from human cells that have not been described before. Following the convention of the already present human ASM transcripts in GenBank, the new transcripts were termed ASM-5 (GenBank Accession Number HQ132746), ASM-6 (HQ132747) and ASM-7 (HQ132748) (Figure 1).

ASM-5 was isolated from human neuroglioma cells. It lacks the first 69 bp of exon 3 (relative to ASM-1), which results in a reduced transcript length of 1818 bp (Figure S1). At the protein level, the deletion disrupts the catalytic domain of the putative enzyme (606 aa; theoretical molecular weight of 67 kDa) as amino acids 362 to 384 are absent from the protein (Figure S2). ASM-6, identified in human neuroglioma cells, is characterised by a 20 bp intronic insertion derived from the end of intron 5 proximal to exon 6 (exon 5a). This generates a transcript of 1907 bp. The intronic insertion creates a frameshift in the coding sequence. The frameshift introduces a premature stop codon (TAG) and results in a truncated open reading frame of 1521 bp (Figure S1). Consequently, the putative protein (506 aa; theoretical molecular weight of 56 kDa) displays only a partial C-terminal domain in which the terminal 136 amino acids of ASM-1 have been lost and replaced by a unique peptide of 13 amino acids (VSPTSLQVTVCTK) (Figure S2). ASM-7 was identified in human PBMCs and contains a 40 bp intronic sequence derived from the beginning of intron 2 proximal to exon 2 (exon 2a). The resulting transcript is 1921 bp in length. This intronic insertion creates a frameshift and a premature stop codon (TGA), resulting in an open reading frame of 1197 bp (Figure S1). The putative enzyme (398 aa; theoretical molecular weight of 45 kDa) displays only a fragment of the catalytic domain and is devoid of its carboxy-terminal domain because it lacks the C-terminal 267 amino acids. In its place, it carries a unique carboxy-terminal peptide of 38 amino acids (YLSSVETQEGKRKNWGVL-CSFPIPRSPPHLSQYEFLFP) (Figure S2). For an overview see Table 1.

**Table 1:** Characteristics of the newly identified ASM splice variants.

ASM variant	Accession No.	Alternative splicing event	Affected exon	Orthologous transcripts	Impact on protein structure
ASM-5	HQ132746	Acceptor downstream of exon 2	Exon 3	-	Disruption of catalytic domain
ASM-6	HQ132747	Intron between exon 5 and 6	Exon 6	*Pan troglodytes*	Disruption of C-terminal domain;
				*Macaca mulatta*	Specific peptide
ASM-7	HQ132748	Intron between exon 2 and 3	Exon 2	*Pongo abelii*	Disruption of catalytic and
					C-terminal domain; Specific peptide

Exon numbers are based on the full-length ASM-1 transcript.

An *in silico* examination of conservation among orthologues revealed that ASM-6 displays the same combination of splicing events as orthologous ASM transcripts from *Pan troglodytes* (XM_001164317.1) and *Macaca mulatta* (XM_001110020.1). ASM-7 exhibits a combination of splicing events identical to that of the orthologous ASM transcript expressed in *Pongo abelii* (NM_001132129.1). No orthologous transcript contained the type of splicing event identified in ASM-5 (Table 1).

In a second screen on human brain tissue, 28% of the 149 analysed ASM transcripts had undergone alternative splicing. These data confirm the high frequency of alternative splicing seen in our first screen results. The splicing events that occur in ASM transcripts ASM-2 to -7 were detected in various combinations and, thus, may constitute the whole ASM transcriptome.

Thus, ASM is subject to alternative splicing processes, which generate distinct transcripts through a defined set of splicing events. These splicing events are likely to be highly conserved.

### ASM Splicing Patterns Vary in Human Tissues

**Figure 2:**
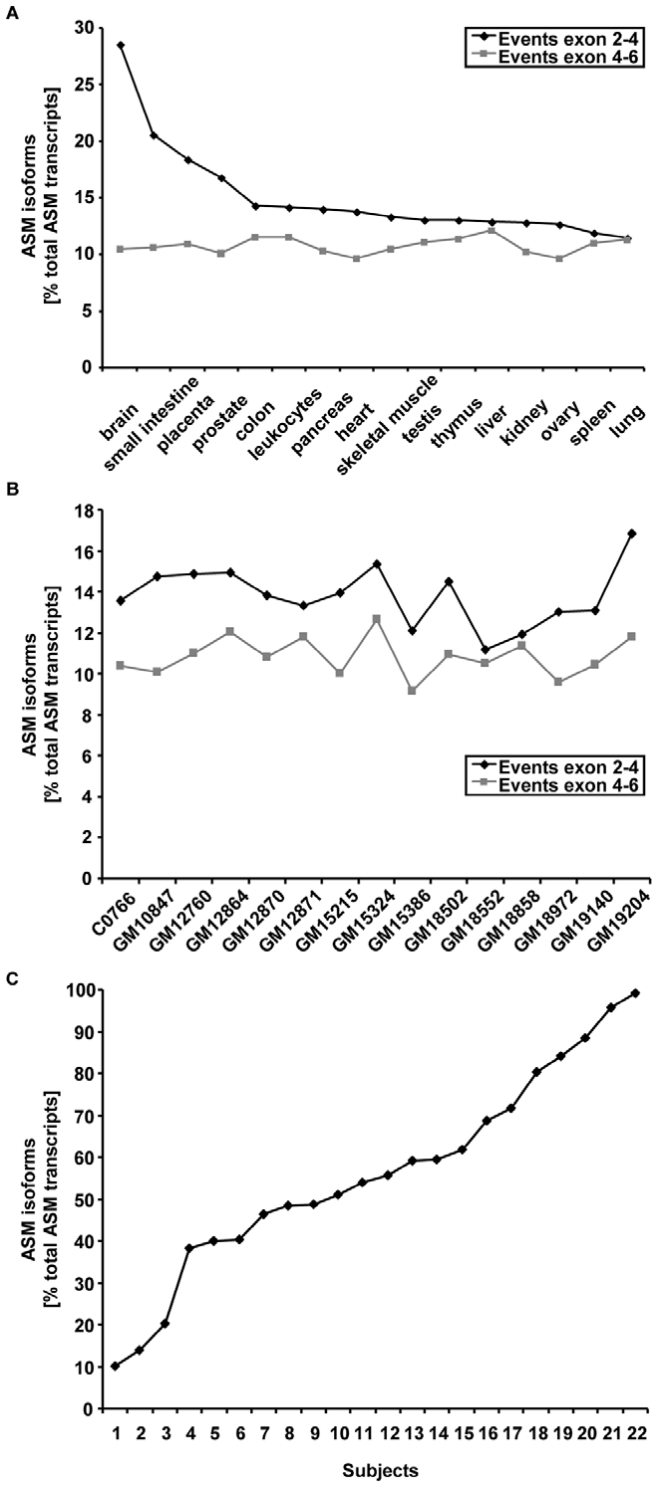
Extent of tissue-specific ASM isoform expression. **A. Human tissues.** ASM isoform expression varied tissue-specifically, depending on the type of splicing event. Isoform fractions derived from splicing events between exons 2 and 4 showed high levels of variation, with brain tissue expressing the highest percentage of ASM isoforms. Isoform fractions derived from splicing events between exons 4 and 6 showed low levels of variation. Capillary electrophoresis with laser-induced fluorescence analysis was conducted on triplicate RNA isolations of different human tissues. Data indicate ASM isoform percent fractions of splicing events occurring between exons 2 and 4 and exons 4 and 6. **B. Human lymphoblastoid cell lines.** ASM isoform expression in human lymphoblastoid cell lines revealed a low level of inter-individual variation for splicing events occurring between exons 2 and 4 and exons 4 and 6. Capillary electrophoresis with laser-induced fluorescence analysis was conducted on triplicate RNA isolations. Data indicate ASM isoform percent fractions of splicing events occurring between exons 2 and 4 and exons 4 and 6. **C. Human primary blood cells.** ASM isoforms were expressed in each of the 22 healthy individuals’ blood cell RNA, but the relative contribution of ASM isoforms to the total amount of ASM transcripts varied highly between subjects. Capillary electrophoresis with laser-induced fluorescence analysis was conducted. Data indicate ASM isoform percent fractions of splicing events occurring between exons 2 and 6.

To explore the tissue-specific expression of ASM splice variants, we analysed all splicing events in the splicing-relevant parts of ASM using fluorescence-based quantification of electrophoretically separated RT-PCR products. ASM isoform ratios were determined for 16 different human tissues and comprised all splicing events between exons 2 and 4 and exons 4 and 6. Isoform fractions derived from splicing events between exons 2 and 4 varied between 11% and 14%, showing a low level of variation between tissues. Of note, four tissues were found to express much higher levels of alternatively spliced transcripts: the brain (29% of total ASM transcripts), the small intestine (21%), the placenta (18%) and the prostate (17%). Isoform fractions between exons 4 and 6 accounted for 10–12% of all ASM transcripts, showing low levels of variation (CV of 7%) (Figure 2A). Thus, ASM alternative splicing varies by tissue, with more variation in splicing events between exons 2 and 4.

For the determination of inter-individual variation in ASM alternative splicing, we investigated the RNA of human lymphoblastoid cell lines, which were derived from human donors. Isoform fractions showed a low level of inter-individual variation in splicing events occurring between both exons 2 and 4 (14–17% of total ASM transcripts, CV of 11%) and exons 4 and 6 (9–13%, CV of 9%) (Figure 2B). ASM alternative splicing therefore seems to be constant in human B-lymphocyte cell lines.

To test the inter-individual variation of ASM splicing in human primary cells, we conducted an analysis on whole-blood RNA from 22 healthy donors. Surprisingly, the relative contribution of ASM isoforms to the total number of ASM transcripts varied highly between subjects (10–99%, CV of 44%) (Figure 2C). This high level of variation between subjects was also observed during an analysis of these RNA samples using RT-qPCR for variants ASM-1 and ASM-5 to -7 (Data not shown). ASM alternative splicing in primary blood cells is highly different between individuals and thus seems to be context-dependent and under some regulatory control.

### New ASM Splice Variants are Catalytically Inactive

**Figure 3:**
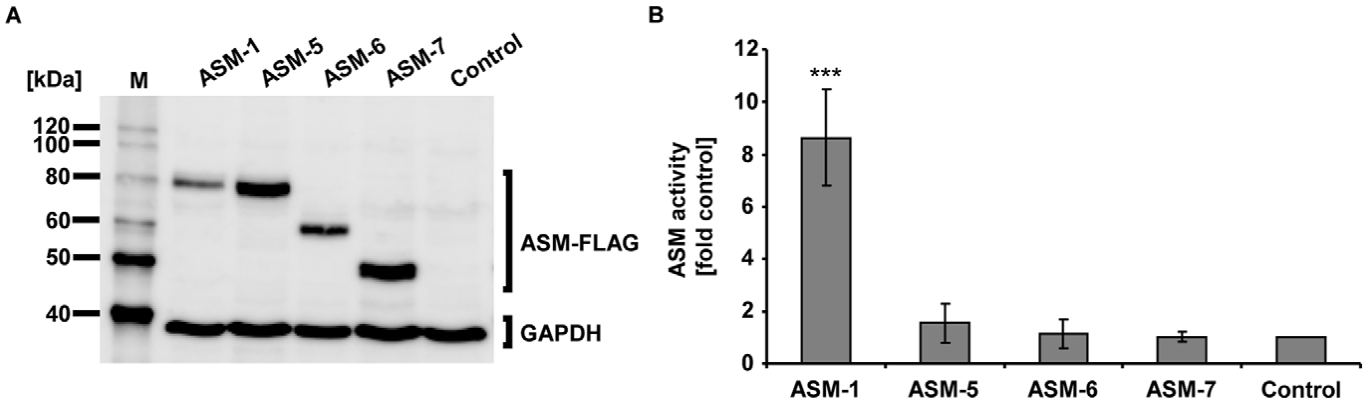
Biochemical characterisation of ASM-1 and the novel isoforms ASM-5, -6 and -7. A. Expression of ASM-FLAG constructs in HeLa cells. ASM-1 and ASM-5 to -7 produced proteins that corresponded to their predicted sizes. Lysates were analysed by Western blotting 48 h after transfection. ASM-FLAG variants were detected using an anti-FLAG antibody. The cloning vector served as a negative control; GAPDH was used as a loading control. M indicates the size marker. **B. Novel variants ASM-5 to -7 are catalytically inactive.** ASM-1 overexpression significantly increased ASM activity relative to the vector control (p<0.001), in sharp contrast to the novel isoforms ASM-5, -6 and -7. Lysates were subjected to an *in vitro* enzyme activity assay 48 h after transfection of H4 cells with 7.5 µg of the respective ASM-FLAG construct. The results are provided as the fold increase in ASM activity relative to the control (set to 1). Bars indicate mean values of n = 8 independent experiments; error bars indicate SD. Statistical significance was calculated with respect to the control using a one-sampled t-test (***p<0.001).

To analyse the intrinsic biochemical properties of ASM splice variants resulting from typical splicing events in more detail, the respective cDNAs were cloned into expression vectors. Western blot analyses showed that ASM-1 and each of the new splice variants ASM-5 to -7 were translated into protein upon overexpression and displayed the predicted sizes (Figure 3A).

An *in vitro* enzymatic assay using radiolabelled C12-sphingomyelin as substrate [24] revealed that ASM-1 overexpression in H4 cells significantly increased ASM activity in cell lysates by 9-fold over endogenous levels (63.4±22.5 pmol/h/µg (overexpressed ASM activity) versus 8.1±5.5 pmol/h/µg (endogenous ASM activity); p<0.001; n = 8). In contrast to ASM-1, none of the alternatively spliced variants displayed catalytic activity that was above endogenous levels (Figure 3B), despite easily detectable expression of each of the proteins. To assess the impact of the C-terminal tag, we expressed variants without the FLAG-tag, but this did not alter the results. Similar results were obtained when the experiments were repeated with HeLa and HEK293 cell lines, varying cell numbers, five different plasmid preparations and expression constructs with a different epitope tag. The results were confirmed by an alternative ASM activity assay involving a fluorescent C12-sphingomyelin substrate and thin layer chromatography (Data not shown).

We concluded that the new ASM isoforms are catalytically inactive, irrespective of whether they retain the catalytic domain or not.

### New ASM Splice Variants Act in a Dominant-negative Manner upon Overexpression

**Figure 4:**
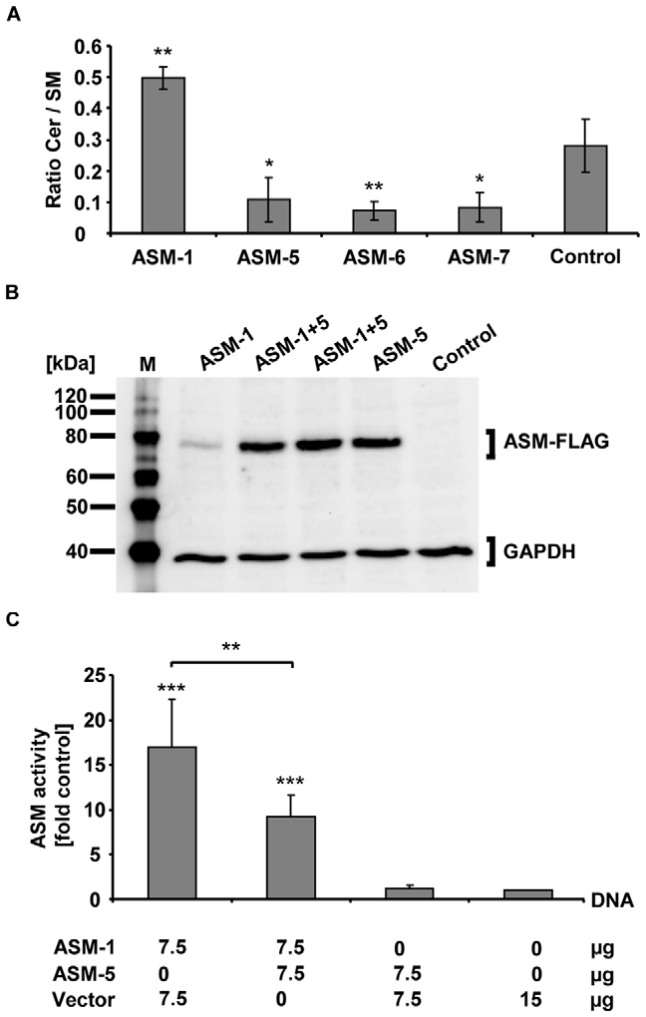
New ASM splice variants act in a dominant-negative manner. A. Modulation of ceramide:sphingomyelin ratios. The ratio of ceramide levels to sphingomyelin levels significantly increased after ASM-1 overexpression (p<0.01). In contrast, the ratio significantly decreased upon overexpression of ASM-5, -6 and -7 (p<0.01 for ASM-6; p<0.05 for ASM-5 and -7). Lysates were subjected to MALDI-TOF MS for sphingolipid analysis 24 h after transfection of H4 cells. The data indicate mean values of n = 3 independent experiments; error bars indicate SD. Statistical significance was calculated with respect to the control using the t-test (*p<0.05, **p<0.01). **B. Co-expression of ASM-FLAG constructs in HeLa cells.** ASM-1 and ASM-5 produced proteins that corresponded to their predicted sizes independent of single- or co-transfection. For control reasons lysates derived from two independent co-transfections were used. Lysates were analysed by Western blotting 72 h after transfection. ASM-FLAG variants were detected using an anti-FLAG antibody. The cloning vector served as a negative control; GAPDH was used as a loading control. M indicates the size marker. **C. ASM-5 exerts a dominant-negative effect on ASM-1.** ASM-1 overexpression generated high ASM activity relative to a vector control (p<0.001). Co-expression of ASM-1 and ASM-5 at a ratio of 1:1 led to a significant reduction in ASM activity relative to conditions where ASM-1 was expressed alone (p<0.01). ASM activity levels of this co-expression were still above a vector control (p<0.001). HeLa cells were transfected with 15 µg of ASM-FLAG constructs and cultured for 48 h. After 24 h of serum-starvation lysates were subjected to an *in vitro* enzyme activity assay. The results are presented as the fold increase in ASM activity relative to a negative control. The displayed data represent mean values of n = 6 independent experiments; error bars denote SD. Statistical significance was calculated using one-way ANOVA including a Bonferroni post-hoc test (**p<0.01, ***p<0.001).

Based on the intrinsic properties of the new ASM isoforms, their role for sphingolipid metabolism *in vivo* was more specifically defined. We examined to what extent the new ASM isoforms modulate the physiological occurring ceramide and sphingomyelin species levels (Table S1) using MALDI-TOF MS analysis. Overexpression of ASM-1 significantly increased the ratio of ceramide to sphingomyelin in H4 cells by 1.8-fold compared to the control (p<0.01; n = 3). This is consistent with the assumption that increased ASM activity results in increased conversion of sphingomyelin to ceramide. In contrast, transfection of the new ASM splice variants had the opposite effect. The ceramide to sphingomyelin ratio decreased to 26–38% of the control (p<0.01 for ASM-6; p<0.05 for ASM-5 and -7) (Figure 4A). Thus, the novel ASM splice variants appear to exert an inhibitory effect on cellular sphingomyelin hydrolysis *in vivo*.

The modifying impact of the novel ASM splice variants on ASM activity was further investigated in co-transfection experiments, where the novel human and non-conserved splice variant ASM-5 was co-transfected with ASM-1. Western blotting indicated that both ASM-5 and ASM-1 were expressed (Figure 4B). Expression of ASM-1 alone caused a significant increase in ASM activity over endogenous levels (p<0.001; n = 6), whereas expression of ASM-5 alone did not result in increased ASM activity. Of note, co-transfection of ASM-5 with ASM-1 significantly reduced ASM activity compared to ASM-1 overexpression alone (9-fold vs. 17-fold over endogenous levels; p<0.01) (Figure 4C). This implies a dominant-negative effect of ASM-5 on ASM-1 activity.

### Physiological Cell Models Confirm the Dominant-negative Effect of New ASM Splice Variants

**Figure 5:**
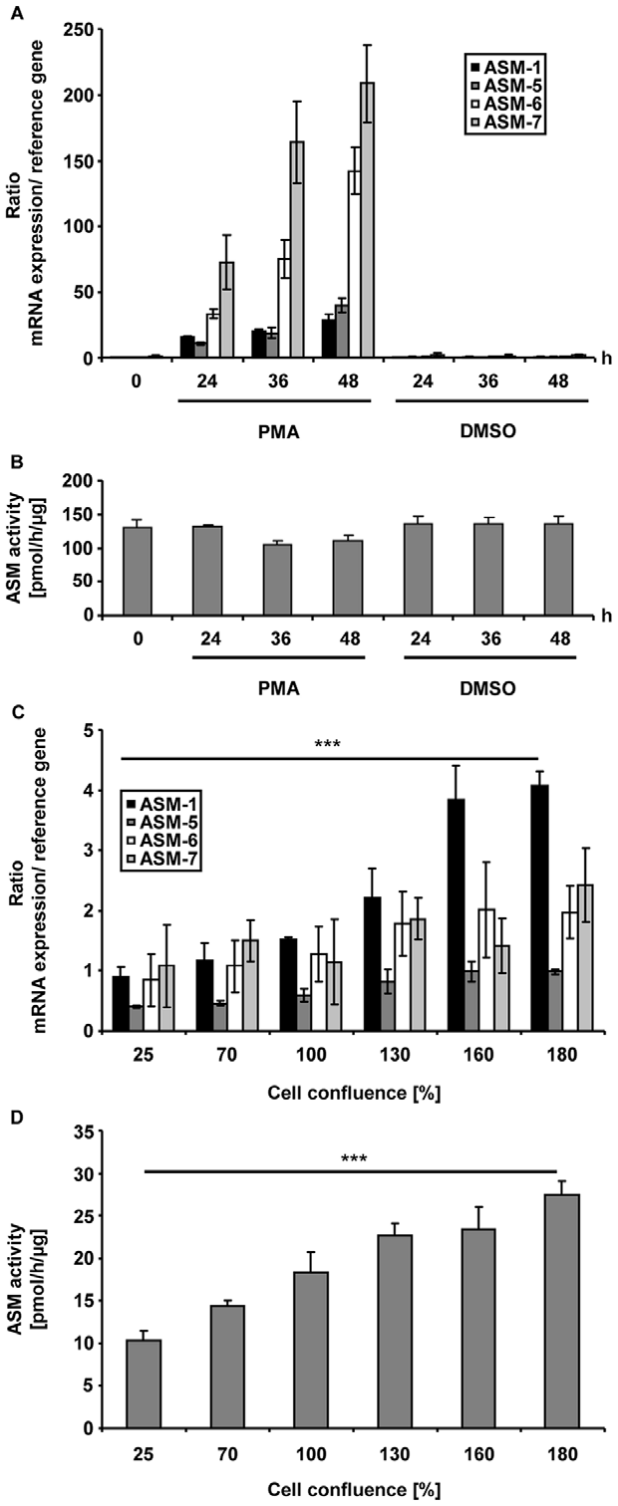
Physiological cell models confirm the dominant-negative effect of new ASM splice variants. **A. PMA induces mRNA expression of ASM isoforms in THP-1 cells.** Upon PMA treatment, ASM-1 mRNA expression was time-dependently induced. Expression levels of ASM-5 to -7 rose disproportionately strong. THP-1 cells were treated with 160 nM PMA. After 24, 36 and 48 h, quantitative RT-PCR analyses were conducted. All ASM RT-qPCR values were measured in duplicates, normalised against values from the non-regulated reference gene *HPRT* and included primer efficiency calculations. DMSO served as a negative control. The displayed data represent mean values of triplicates, error bars denote SD. The results were replicated three times. **B. ASM activity levels remain constant upon PMA treatment of THP-1 cells.** Upon 24, 36 and 48 h of PMA treatment (160 nM), cell lysates were subjected to an *in vitro* enzyme activity assay. DMSO served as a negative control. The displayed data represent mean values of triplicates, error bars denote SD. Results were replicated three times. **C. High density of H4 cells induces mRNA expression of full-length ASM-1.** Depending on increasing cell density, ASM-1 mRNA expression was significantly induced (p<0.001), in contrast to the expression of splice variants ASM-5, -6 and –7. H4 cells were grown to varying levels of confluence. After 72 h, quantitative RT-PCR analyses were conducted. All ASM RT-qPCR values were measured in duplicates and normalised against values from the non-regulated reference gene *HPRT*. The displayed data represent the mean values of three independent experiments, error bars denote SD. Statistical significance was calculated using one-way ANOVA including a post-hoc test for linear trend (*** p<0.001). **D. High density of H4 cells increases ASM activity.** The increase in cellular density significantly increased cellular ASM activity (p<0.001). H4 cells were grown to varying levels of confluence. Upon 72 h cell lysates were subjected to an *in vitro* enzyme activity assay. The displayed data represent the mean values of three independent experiments, error bars denote SD. Statistical significance was calculated using one-way ANOVA including a post-hoc test for linear trend (*** p<0.001).

To determine the biological relevance of alternatively spliced ASM, we investigated the dominant-negative effect in physiological cell models. THP-1 cells treated with the phorbol ester PMA serve as a well-established model for monocyte differentiation. During monocyte to macrophage differentiation, transcription of ASM is upregulated [15]. THP-1 cells were treated with PMA and analysed at different time points. Expression levels of each novel splice variant were analysed using quantitative RT-PCR and correlated with the respective cellular ASM activity levels. Upon PMA treatment, ASM-1 mRNA expression was time-dependently induced, which cumulated in 36-fold expression after 48 h. However, elevated ASM-1 mRNA expression was accompanied by a disproportionately strong increase in the mRNA of the novel ASM splice variants (up to 200-fold) (Figure 5A). Of note, despite the clear increase in ASM-1 mRNA, ASM activity levels remained unchanged upon PMA treatment (Figure 5B). This is consistent with the assumption that an increased expression of ASM isoforms prevents an increase in ASM activity. Thus, the dominant-negative effect exerted by novel ASM splice variants may play an important role during monocyte to macrophage differentiation.

A further physiological cell model deals with the well-known phenomenon that sphingolipid metabolism significantly differs depending on cell confluence levels [25]. H4 cells were grown to varying levels of confluence and analysed for expression levels of each novel splice variant and their respective cellular ASM activity levels. ASM-1 mRNA expression was low in preconfluent H4 cells, but significantly increased when cells became confluent or over-confluent (4-fold; p<0.001; n = 3). In contrast, expression of new splice variants ASM-5, -6 and -7 rose 2-fold at most (Figure 5C). As ASM-1 mRNA expression significantly increased at cellular confluence, so did ASM activity levels (2.7-fold; p<0.001; n = 3) (Figure 5D). In this model, the novel splice variants were not significantly induced. Analogously, the confluence-mediated increase in ASM-1 mRNA expression resulted in a significantly increased ASM activity level. This indirectly stresses the influence of ASM splice variants on cellular ASM activity.

## Discussion

In this study, we identified three new alternatively spliced human ASM transcripts. Contrary to the previously described finding that ASM-1 constitutes 90% of the ASM transcripts and alternatively spliced variants 10% [22], [23], we detected a much higher frequency of alternative splicing events for ASM in our screens. This difference in splicing frequencies may be due to the different tissues under investigation. Former studies analysed cDNA libraries of fibroblast, placental and testis tissue, while our study screened neuronal cells and blood cells. In our quantitative analysis of further tissues we found that there exist five tissues, which constitute an exception to the 10% alternative splicing level: the brain, the small intestine, the placenta, the prostate and blood cells. It should be noted that alternative splicing is particularly important for neuronal functions [26] and that the brain exhibits the highest number of tissue-specific splice variants [27]. This is in line with our analysis of tissue-specific ASM splicing because brain tissue comprised 30% of alternatively spliced ASM variants.

A second important point is the considerable influence of external stimuli such as cellular stress on splice site selection [28]. Importantly, the main features of the mammalian stress response include changes in the peripheral and central nervous systems and modifications to blood cell composition. For example, stress-induced alternative splicing of the acetylcholinesterase enzyme occurs selectively in brain and blood cells [29]. The alternative splicing of ASM may be analogous: tissue dependent and particularly abundant in brain and blood cells as part of the cellular stress response. This hypothesis is reinforced by our results regarding the splicing patterns in human primary blood cells and in the monocyte cell line THP-1. In both cases, the variation levels of ASM splicing are very high. In the THP-1 model, alternative splicing varies depending on external stimulation by PMA, which in turn has influence on ASM activity. Since ASM activity in blood cells fluctuates and is influenced by diverse factors, also alternative splicing in blood cells could be influenced, explaining the high inter-individual differences. Due to the constant splicing levels in B-lymphoblasts in our experiments and the importance of ASM for macrophage biology [30], it seems likely that the varying splicing patterns in primary blood cells result selectively from macrophages and are triggered by the specific physiological context. In future studies, splicing patterns for separated blood cell populations should be monitored.

Because ASM plays an essential role in the fragile balance of the rheostat, ASM alternative splicing is expected to occur within a highly regulated context. There seems to be only a limited number of splicing events that generate the alternatively spliced ASM transcripts. These splicing events and even their specific combinations are conserved among different species. For example, the 40 bp intronic sequence from intron 2 found in ASM-7 is identical to a splicing event in ASM-2 and to certain orthologues in other species. Similarly, the 20 bp intronic sequence from intron 5 in ASM-6 is the same as one of the events in transcript ASM-4 and its orthologues. The splicing event that generates transcript ASM-5 seems to be unique for humans thus far; there are no equivalent motifs present in ASM transcripts from other species. It is under discussion that the brain-related diversification between primates and humans occurred as a result of an increase in alternative splicing [31], [32]. In this context, ASM-5 might contribute specifically to the regulation of the human stress response.

The modification of protein properties as a result of alternative splicing, such as an alteration to the intrinsic catalytic property of an enzyme, is a well-documented phenomenon [33], [34], [35]. A switch from enzymatic activity to inactivity can be triggered by stress signals via the mechanism of alternative splicing [28]. Full-length ASM-1 consists of a regulatory saposin-B domain, a catalytic domain and a C-terminal domain. The C-terminal domain is thought to mediate ASM dimerisation [20]. The proteins generated by the alternative splicing of ASM that have been studied so far are catalytically inactive. Not all, however, are characterised by a disrupted catalytic domain. For ASM-6, the disruption of the C-terminal domain generates a catalytically-inactive ASM isoform. Thus, it would appear that the C-terminal domain plays an essential role in ASM function. This is supported by analyses of SNPs associated with Niemann-Pick disease type A and B. The SNPs that cause the loss of ASM function are located within the coding sequences for the catalytic but also for the C-terminal domain [36], [37], [38], [39], [40], [41], [42], [43], [44]. The dominant-negative isoform ASM-5 retains all domains present in ASM-1 except a small portion of the catalytic domain. Thus, the dominant-negative effect could be mediated via the dimerisation of ASM-1 with ASM-5 which seems to be augmented in serum-starving conditions. The dominant-negative mode of action of ASM-5 may be an important step in preventing an induced activation of ASM-1, which would otherwise lead to ceramide accumulation and ceramide-induced responses. These processes could help to regulate cellular homeostasis. A good example of a physiological role for this effect is given by our THP-1 experiments. Despite clearly elevated ASM-1 mRNA levels during differentiation (see also [15]), ASM activity levels remained constant, possibly due to the strong induction of ASM-5 to -7 mRNA. This could resemble an important phase during monocyte to macrophage differentiation, where the accumulation of further lipids additional to cholesterol is crucial for the cell [45]. An inverse situation is seen in our cell density experiments, where the increase in ASM-1 mRNA expression is accompanied by an increased cellular ASM activity level. Here, none of the modulating effects of new ASM isoforms are observed, as their mRNA expression remains nearly constant. These data stress the fact that sphingolipid metabolism is significantly influenced by cell confluence levels [25]. For sphingolipid research using cell culture models it is of highest importance to consider these influencing factors. It is not resolved, however, if these physiological effects are mediated on message or protein level because the detection of endogenous ASM and its isoforms in Western blot analyses is currently difficult. But taking together, dominant-negative effects in sphingomyelin hydrolysis could comprise important regulative processes in physiological settings.

In conclusion, our study identified three novel human alternatively spliced ASM transcripts: ASM-5, ASM-6 and ASM-7. These transcripts show differential expression patterns in the blood cells of healthy individuals and code for enzymes that are catalytically inactive *in vitro*. We were able to show for the first time that inactive ASM variants can have functional consequences for cellular processes. We would like to suggest that the alternative splicing of ASM could be of great importance to the rebalancing of cellular ASM activity after exposure to stress stimuli. Further studies are being undertaken to elucidate the role of alternative splicing in neuro-psychiatric diseases linked to ASM.

## Materials and Methods

### Ethics Statement

The collection of blood samples was approved by the Ethics Committee of Friedrich-Alexander-University of Erlangen-Nuremberg and conducted in concordance with the Declaration of Helsinki. Written informed consent was obtained from all participants.

### Identification and Cloning of ASM Transcripts

Total RNA from peripheral blood mononuclear cells (PBMCs) obtained by Ficoll density gradient centrifugation (Biocoll Separation Solution, Biochrom, Berlin, Germany) of the blood from four healthy volunteers and from the human neuroglioma cell line (H4) was isolated using QIAzol lysis reagent (Qiagen, Hilden, Germany) in accordance with the manufacturer’s recommendations. The concentration of RNA was determined photometrically using a Nanodrop spectrophotometer (Peqlab, Erlangen, Germany), and RNA integrity was assessed by nondenaturing agarose gel electrophoresis and capillary gel electrophoresis (Experion, Bio-Rad, Munich, Germany). cDNA was synthesised using the VILO cDNA reaction kit (Invitrogen, Darmstadt, Germany) in a reaction volume of 20 µl with 1 µg of total RNA as a template. Full-length ASM transcripts were amplified by polymerase chain reaction (PCR) in a total reaction volume of 50 µl with 1 µl of undiluted cDNA, 1 U KAPA HiFi DNA polymerase in GC-buffer, 1.7 mM MgCl_2_, 0.3 mM dNTPs (Peqlab, Erlangen, Germany) and 0.24 µM oligonucleotides (Operon, Ebersberg, Germany). Primer sequences were based on the reference sequence of ASM-1. For the PCR, primers carrying *Bsp*E1 and *Bgl*II restriction sites for subsequent cloning were designed to anneal to regions flanking the initiation and stop codons of ASM-1 (Table 2). Cycle parameters were as follows: an initial denaturation step at 95°C for 2 min, 35 cycles at 98°C for 20 sec, 65°C for 15 sec, 68°C for 1 min, and a final extension step at 68°C for 5 min. In a second approach, RT-PCR was performed on brain tissue cDNA (Multiple Tissue cDNA, Clontech, Mountain View, CA) using BioMix white (Bioline, Randolph, MA) in a total volume of 25 µl, including 3 µl cDNA and 10 pmol oligonucleotides (fw 5′-ccttcattgagggcaaccac-3′; rev 5′-ggtatgtttgcctgggtcag-3′). Cycle parameters were as follows: initial denaturation at 94°C for 5 min, 30 cycles at 94°C for 30 sec, 58°C for 30 sec, 72°C for 1 min, and a final step at 72°C for 10 min.

**Table 2:** Oligonucleotides used for cloning of ASM transcripts.

ASM transcript	Forward sequence 5' -> 3'	Reverse sequence 5' -> 3'	Annealing
ASM-1; ASM-5	tccctccggaatgccccgctacggagc	gcgagatctgcaaaacagtggccttgg	65°C
ASM-6	tccctccggaatgccccgctacggagc	ggaagatctacgggaacaaaaattcatatgaagagagagg	65°C
ASM-7	tccctccggaatgccccgctacggagc	ggaagatctcgggaacaaaaattcatattgagagagatg	65°C

PCR products were purified using the QIAquick PCR purification kit (Qiagen, Hilden, Germany) and cloned for analysis using the StrataClone Blunt PCR cloning kit (Agilent, Waldbronn, Germany). Amplified products were analysed by sequencing and compared to the reference sequence of ASM-1.

### Quantification of ASM Splicing Events

#### PCR amplification and capillary electrophoresis with laser-induced fluorescence analysis

Total RNA from human lymphoblastoid cell lines was isolated over three consecutive days using the RNeasy Mini Kit (Qiagen, Hilden, Germany) according to the manufacturer’s protocol. Human lymphoblastoid cell lines were Epstein-Barr virus (EBV)-transformed lymphoblastoid cells cultured in RPMI 1640 medium supplemented with 15% FCS and 2 mM L-glutamine (Gibco, Eggenstein, Germany) at 37°C, 5% CO_2_ and 95% humidity. The human cell lines GM10847, GM12760, GM12864, GM12870, GM12871, GM15215, GM15324, GM15386, GM18502, GM18552, GM18858, GM18972, GM19140, and GM19204 were obtained from the Coriell Cell Repository (Camden, NJ) and C0766 from the European Collection of Cell Cultures (Salisbury, UK). cDNA synthesis was performed with the Sprint RT Complete-Random Hexamer first-strand cDNA synthesis kit (Clontech-Takara Bio Europe, Saint-Germain-en-Laye, France) according to the manufacturer’s protocol. 5 µg of total RNA were used for reverse transcription. Total RNA from the blood of healthy volunteers was collected using the PAX system (Qiagen, Hilden, Germany). A half µg of RNA was used in a 20 µl reverse transcription reaction (using components of the VILO cDNA reaction kit (Invitrogen, Darmstadt, Germany)) to synthesise cDNA.

RT-PCR was performed on the cDNA of human lymphoblastoid cells, human blood cells and human tissue (Multiple Tissue cDNA Panels I and II, Clontech, Mountain View, CA) with BioMix white (Bioline, Randolph, MA) in a total volume of 25 µl, including 3 µl of cDNA and 10 pmol of oligonucleotides (Table 3). Cycle parameters were as follows: initial denaturation at 94°C for 5 min, 30 cycles at 94°C for 30 sec, 58°C for 30 sec, 72°C for 1 min, and a final step at 72°C for 10 min. Capillary electrophoresis with laser-induced fluorescence analysis was conducted as described in [46].

**Table 3:** Oligonucleotides used for RT-PCR and subsequent capillary electrophoresis.

ASM exons	Forward sequence 5' -> 3'	Reverse sequence 5' -> 3'	Annealing
Exon 2 to 4	FAM-tcctggggccagtgccag	cagctcttcagacagtgcc	58°C
Exon 4 to 6	FAM-caggatgtaggtctcatggtc	gctggagctggaattattacc	58°C
Exon 2 to 6	FAM-ccttcattgagggcaaccac	ggtatgtttgcctgggtcag	58°C

#### Quantitative RT-PCR of ASM splice variants

Total RNA using the High Pure RNA Isolation Kit (Roche, Mannheim, Germany) was collected from human acute monocytic leukaemia THP-1 cells provided by M. Lehner [47], which were cultured in RPMI 1640 medium supplemented with 10% (v/v) FCS and 2 mM L-glutamine and differentiated using 160 nM phorbol 12-myristate 13-acetate (PMA; Sigma-Aldrich, Munich, Germany). Total RNA from human neuroglioma H4 cells (Promochem, Wesel, Germany), cultured in DMEM medium supplemented with 10% (v/v) FCS and 4 mM L-glutamine, was collected using QIAzol lysis reagent (Qiagen, Hilden, Germany) in accordance with the manufacturer’s recommendations. The concentration of RNA was determined photometrically using a Nanodrop spectrophotometer (Peqlab, Erlangen, Germany) and RNA integrity was assessed by nondenaturing agarose gel electrophoresis and capillary gel electrophoresis (Experion, Bio-Rad, Munich, Germany). A half µg of RNA was used in a 20 µl reverse transcription reaction (using components of the VILO cDNA reaction kit (Invitrogen, Darmstadt, Germany)) to synthesise cDNA.

RT-qPCR analyses were performed in a reaction volume of 10 µl with the SYBR green I master mix (Roche, Mannheim, Germany), 2.5 µl of diluted cDNA and HPLC-purified oligonucleotides at a final concentration of 1 µM each (Operon, Ebersberg, Germany) (Table 4) in white multiwell plates (Roche, Mannheim, Germany). Oligonucleotides were tested for target-specificity (Figure S3). Cycle parameters were as follows: initial denaturation at 95°C for 5 min, 40 cycles at 95°C for 10 sec, ASM variant-specific primer annealing temperatures for 20 sec and 72°C for 20 sec. Expression levels of ASM transcripts were normalised against RT-qPCR values for the invariant reference gene *HPRT* (NM_000194.2). Data were analysed with the LightCycler® 480 software (Roche, Mannheim, Germany), which provides algorithms for an advanced relative quantification method that includes calibrator normalising and the calculation of primer efficiency for each target. Melting curves were monitored and amplification products were verified by agarose gel electrophoresis (1%) and subsequent nucleotide sequencing. The RT-qPCR analyses were performed in accordance with MIQE guidelines [48]. For further details on the analyses, see Table S2.

**Table 4:** Oligonucleotides used for RT-qPCR analyses.

Transcript	Forward sequence 5' -> 3'	Reverse sequence 5' -> 3'	Amplicon (bp)	Annealing
ASM-1	cctcagaattggggggttctatgc	cacacggtaaccaggattaagg	412	62°C
ASM-5	tgcagacccactgtgctgc	aagttctcacgggaactgagggtgcgcagg	440	68°C
ASM-6	gctgcctgccgaagccctgc	gcaaggatgtggggctgacagg	440	68°C
ASM-7	tgcagacccactgtgctgc	accccccaattctttcttttccc	474	68°C
HPRT	tccgcctcctcctctgctc	gaataaacaccctttccaaatcctca	185	62/68°C

### Overexpression Studies on ASM Isoforms

#### Construction of expression plasmids

Open reading frames were amplified from the plasmids into which the initial PCR products were cloned using a single forward primer and reverse primers specific for different truncated variants (Table 2). Amplimers were digested with the *Bsp*E1 and *Bgl*II restriction endonucleases and inserted between *Xma*I and *Hind*III sites in the FLAG-N2 expression vector to create an in-frame C-terminal fusion of various ASM gene products with the 16 amino acid FLAG-tag. Each inserted sequence was verified by sequence analysis.

#### Cell Culture

H4, HeLa and HEK293 cells (Promochem, Wesel, Germany) were cultured in DMEM medium supplemented with 10% (v/v) FCS and 4 mM (for H4 cells) or 2 mM L-glutamine (for HeLa and HEK293 cells). Cells were maintained at 37°C in a humidified atmosphere with 8.5% CO_2_ and were monitored for potential mycoplasma infection using the MycoAlert Mycoplasma Detection Kit (Lonza, Cologne, Germany). All reagents used for cell culture were purchased from Biochrom (Berlin, Germany).

For transfection purposes, cells were grown to 60% confluence in 6-well plates with 2 ml of culture medium. Transfections were performed by the calcium phosphate precipitation procedure. 7.5 µg of plasmid DNA were mixed with 2x N,N-Bis(2-hydroxyethyl)-2-aminoethanesulfonic acid (BES) buffer [50 mM BES, 280 mM NaCl, 1.5 mM Na_2_HPO_4_, pH 6.98 (for H4 and HeLa cells) and pH 7.01 (for HEK293 cells); all chemicals were obtained from Sigma-Aldrich (Munich, Germany)] containing 10 µl 2.5 M calcium chloride, incubated for 20 min at room temperature, and added to cells. The culture medium was changed 17 h after transfection. Transfection efficiency was approximately 70–80%, as determined by the number of GFP-positive cells after a parallel control transfection with the expression vector pmaxFP-green-C (Lonza, Cologne, Germany).

#### Western blot analysis

Transfected cells were lysed in RIPA buffer [50 mM Tris-HCl (pH 7.5), 150 mM NaCl, 1% NP-40, 0.5% sodium deoxycholate, 0.1% SDS (Sigma-Aldrich, Munich, Germany) into which were dissolved complete protease inhibitor tablets from Roche (Mannheim, Germany)]. Cell debris was removed by centrifugation at 10,000 g for 15 min at 4°C. The protein concentration of the cell extracts was determined using the BCA assay (Bio-Rad, Munich, Germany). Sample aliquots were denatured in Laemmli buffer for 5 min [49]. Total protein (10 µg) was separated by 10% SDS gel electrophoresis and transferred to a PVDF-membrane (Millipore, Schwalbach/Ts, Germany). Blots were incubated in 2.5% milk powder (Roth, Karlsruhe, Germany) and 0.1% Tween 20 (Sigma-Aldrich, Munich, Germany) in PBS at 4°C overnight. Subsequently, the blots were incubated for 4 h at room temperature with a primary mouse monoclonal anti-FLAG-antibody (1:1000; Sigma-Aldrich, Munich, Germany), for 1 h with a primary mouse glyceraldehyde-3-phosphate dehydrogenase (GAPDH) antibody (1:200,000); Millipore, Schwalbach/Ts, Germany), and then for 1 h with a secondary goat anti-mouse IgG antibody coupled to horseradish peroxidase (1:10,000; Dianova, Hamburg, Germany). All antibodies were diluted in 0.5% milk powder solution (Roth, Karlsruhe, Germany) and 0.1% Tween 20 in PBS (Sigma-Aldrich, Munich, Germany). Detection was performed using the enhanced chemiluminescence (ECL) Western blotting detection system (Amersham, Munich, Germany) and visualised on a high-sensitivity camera device (FluorSMax, Bio-Rad, Munich, Germany).

#### In vitro determination of ASM activity

Enzymatic ASM activity in the cell extracts was determined as previously described [24]. Quantitative analysis of ASM activity was performed with 10 µg of total cellular protein extract diluted in 250 µl of sodium acetate buffer [250 mM sodium acetate pH 5.0 (Merck, Darmstadt, Germany), 0.1% NP-40 (Sigma-Aldrich, Munich, Germany), 1.3 mM EDTA (Sigma-Aldrich, Munich, Germany) and 1 tablet of complete mini protease inhibitor mix per 10 ml of buffer (Roche, Mannheim, Germany)]. 440 pmol ^14^C-radiolabelled C12-sphingomyelin (Perkin-Elmer, MA, USA) suspended in 30 µl of enzyme buffer [250 mM sodium acetate pH 5.0 (Merck, Darmstadt, Germany), 0.1% NP-40 (Sigma-Aldrich, Munich, Germany), 1.3 mM EDTA (Sigma-Aldrich, Munich, Germany)] was added to the protein extract. The enzymatic reaction was incubated at 37°C for 20 min and then stopped with the addition of 800 µl of chloroform/methanol (2:1, v/v). The two phases were separated by centrifugation. The radioactivity of the ^14^C-labelled product, phosphorylcholine, in the aqueous phase was determined by liquid scintillation counting and was used to calculate ASM activity.

ASM activity was also determined using the fluorescent substrate BODIPY-C12-sphingomyelin (Invitrogen, Darmstadt, Germany). In this assay, 2 µl of the cell extracts (typically corresponding to 0.5-2 µg of protein) were added to 116 pmol of fluorescent substrate in sodium acetate buffer [200 mM sodium acetate pH 5.0, 500 mM NaCl and 0.2% NP-40] in a total volume of 100 µl. After incubation for 0.5 to 4 h at 37°C, the fluorescent product ceramide and the uncleaved substrate were extracted by the addition of 250 µl chloroform/methanol (2:1, v/v). Following vortexing and centrifugation, the organic phase was concentrated in a SpeedVac vacuum concentrator and spotted on silica gel 60 plates (Macherey-Nagel, Düren, Germany). Ceramide and sphingomyelin were separated by thin layer chromatography using chloroform/methanol (4:1, v/v) as a solvent and quantified on a Typhoon Trio scanner (GE Healthcare, 488 nm excitation and 520 nm emission wavelengths, 280 V, 100 µm resolution) with QuantityOne software (Bio-Rad, Munich, Germany). Measurements were performed in triplicates. The enzymatic activity of ASM was calculated as the rate of hydrolysis of sphingomyelin to ceramide per hour and per µg of protein in the cell lysate sample (pmol/h/µg).

#### MALDI-TOF MS analysis of cellular ceramide and sphingomyelin levels

Transfected H4 cells were lysed in 100 µl of lysis buffer [20 mM Tris-HCl pH 7.4, 100 µM butylated hydroxytoluene (spectrophotometric grade, Sigma-Aldrich, Munich, Germany)], immediately frozen in liquid nitrogen and stored at –80°C. After thawing, the cell samples were homogenised and sonicated. The lipid content was subjected to classical chloroform/methanol extraction [50], transferred to a clean tube, dried and resuspended (2%, w/v) in chloroform. Samples were mixed with a matrix consisting of 0.5 M 2.5-dihydroxybenzoic acid in methanol and 0.1% trifluoroacetic acid (spectrophotometric grade, Sigma-Aldrich, Munich, Germany) [51]. Aliquots were spotted on a steel target and subjected to mass spectrometric analysis. Mass spectra were obtained using an Autoflex MALDI-TOF MS (Bruker Daltonics, Bremen, Germany) equipped with a nitrogen laser (λ  = 337 nm). Mass spectra were acquired in reflectron and positive ion modes. Each spectrum consists of an average of 100 laser shots.

Mass-to-charge ratio (m/z) values were compared to the lipid database LIPID MAPS (http://www.lipidmaps.org), according to which the corresponding ceramide and sphingomyelin species were defined for semi-quantitative analysis [52] (Table S1). The relative intensities of both mass signals, [M+H]^+^ and [M+Na]^+^ or [M+K]^+^, were determined and summarised. The ratio of ceramide to sphingomyelin intensities was calculated for each individual spectrum and a mean value was determined by three independent experiments.

### Bioinformatic and Statistical Analyses

*In silico* analyses of ASM orthologous transcripts were performed using tools in the NCBI database. Alignments were generated with the ClustalW alignment software of the European Bioinformatics Institute (www. ebi.ac.uk/clustalw). Protein molecular weights were calculated with the aid of the molecular weight calculator at www.sciencegateway.org/tools/proteinmw.htm. Data were analysed for statistical significance with IBM SPSS Statistics 19 software (SPSS Inc., Chicago, IL, USA).

## Supporting Information

Figure S1 **The coding sequences of full-length ASM-1 and alternatively spliced transcripts ASM-5, -6 and -7 differ at specific locations.** The ASM-5 transcript lacks the first 69 bp of exon 3 and, consequently, has a shorter coding length (relative to ASM-1) of 1818 bp. The ASM-6 transcript is characterised by a 20 bp intronic insertion derived from the end of intron 5. It has a transcript length of 1907 bp. ASM-7 contains a 40 bp intronic sequence derived from the beginning of exon 2, which results in a 1921 bp transcript. All of the transcripts are clearly the result of alternative splicing events because they display sequence identity with the full-length ASM along their lengths, except at the locations described above. Grey portions indicate those sequences that differ from the ASM-1 coding sequence. Stop codons are displayed in bold. The alignment was generated using ClustalW.
(DOC)

Figure S2 **The putative protein sequence of each novel isoform displays specific features.** In comparison with the full-length ASM-1 protein, which consists of 631 amino acids and has a theoretical molecular weight of 70 kDa, ASM-5 is 606 amino acids long and has a theoretical molecular weight of 67 kDa. It carries a disrupted catalytic domain due to the loss of 23 amino acids. ASM-6 constitutes a 506 amino acid protein with a theoretical molecular weight of 56 kDa. It has an intact catalytic domain but lacks the C-terminal domain. Instead, it carries a unique C-terminal peptide composed of 13 amino acids, VSPTSLQVTVCTK. ASM-7 consists of 398 amino acids and has a theoretical molecular weight of 45 kDa. Like ASM-6, it is a smaller protein because of a truncated open reading frame. ASM-7 has a partial catalytic domain and entirely lacks the C-terminal domain of ASM-1. At its C-terminus, it carries a unique peptide of 38 amino acids (YLSSVETQEGKRKNWGVLCSFPIPRSPPHLSQYEFLFP). Specific protein sequences are indicated in grey. The protein domains are coloured differently: yellow, signal peptide (aa 1–48); light blue, saposin-B-domain (aa 91–167); purple, proline-rich-domain (aa 168–200); red, catalytic domain (aa 201–463); green, C-terminal domain (aa 464–631). Mannose-6-phosphate sites (Asn 88, 177, 337, 397, 505, 522) are indicated in light green. The alignment was performed using ClustalW.
(DOC)

Figure S3 **Target-specificity of primer pairs designed for amplification of ASM transcripts.** Amplification of ASM-1 and each novel ASM transcript using the respective primer pair resulted in a specific PCR-product of the expected size separated on a 1% agarose gel. Cloned transcripts were used as templates; water served as a negative control (C). Amplimers were verified by sequence analysis.
(TIF)

Table S1 **MALDI-TOF MS detection of sphingomyelin and ceramide species for semi-quantitative analysis.**
(DOC)

Table S2 **RT-qPCR parameters according to the MIQE précis checklist.**
(DOC)
