# Supplementary material for: Functional Implications of Novel Human Acid Sphingomyelinase Splice Variants
Source: PLoS One. 2012 Apr 27;7(4):e35467. doi: 10.1371/journal.pone.0035467 (PMC3338701; doi:10.1371/journal.pone.0035467)
Supplement: Figure S1 — The coding sequences of full-length ASM-1 and alternatively spliced transcripts ASM-5, -6 and -7 differ at specific locations. The ASM-5 transcript lacks the first 69 bp of exon 3 and, consequently, has a shorter coding length (relative to ASM-1) of 1818 bp. The ASM-6 transcript is characterised by a 20 bp intronic insertion derived from the end of intron 5. It has a transcript length of 1907 bp. ASM-7 contains a 40 bp intronic sequence derived from the beginning of exon 2, which results in a 1921 bp transcript. All of the transcripts are clearly the result of alternative splicing events because they display sequence identity with the full-length ASM along their lengths, except at the locations described above. Grey portions indicate those sequences that differ from the ASM-1 coding sequence. Stop codons are displayed in bold. The alignment was generated using ClustalW. (DOC) [file pone.0035467.s001.doc]

ASM-1 ATGCCCCGCTACGGAGCGTCACTCCGCCAGAGCTGCCCCAGGTCCGGCCGGGAGCAGGGA 60

ASM-5 ATGCCCCGCTACGGAGCGTCACTCCGCCAGAGCTGCCCCAGGTCCGGCCGGGAGCAGGGA 60

ASM-6 ATGCCCCGCTACGGAGCGTCACTCCGCCAGAGCTGCCCCAGGTCCGGCCGGGAGCAGGGA 60

ASM-7 ATGCCCCGCTACGGAGCGTCACTCCGCCAGAGCTGCCCCAGGTCCGGCCGGGAGCAGGGA 60

************************************************************

ASM-1 CAAGACGGGACCGCCGGAGCCCCCGGACTCCTTTGGATGGGCCTGGTGCTGGCGCTGGCG 120

ASM-5 CAAGACGGGACCGCCGGAGCCCCCGGACTCCTTTGGATGGGCCTGGTGCTGGCGCTGGCG 120

ASM-6 CAAGACGGGACCGCCGGAGCCCCCGGACTCCTTTGGATGGGCCTGGTGCTGGCGCTGGCG 120

ASM-7 CAAGACGGGACCGCCGGAGCCCCCGGACTCCTTTGGATGGGCCTGGCGCTGGCGCTGGCG 120

********************************************** *************

ASM-1 CTGGCGCTGGCGCTGGCGCTGGCTCTGTCTGACTCTCGGGTTCTCTGGGCTCCGGCAGAG 180

ASM-5 CTGGCGCTGGCGCTGGC------TCTGTCTGACTCTCGGGTTCTCTGGGCTCCGGCAGAG 174

ASM-6 CTGGCGCTGGCGCTGGC------TCTGTCTGACTCTCGGGTTCTCTGGGCTCCGGCAGAG 174

ASM-7 CTGGCGCTGGC------------TCTGTCTGACTCTCGGGTTCTCTGGGCTCCGGCAGAG 168

*********** *************************************

ASM-1 GCTCACCCTCTTTCTCCCCAAGGCCATCCTGCCAGGTTACATCGCATAGTGCCCCGGCTC 240

ASM-5 GCTCACCCTCTTTCTCCCCAAGGCCATCCTGCCAGGTTACATCGCATAGTGCCCCGGCTC 234

ASM-6 GCTCACCCTCTTTCTCCCCAAGGCCATCCTGCCAGGTTACATCGCATAGTGCCCCGGCTC 234

ASM-7 GCTCACCCTCTTTCTCCCCAAGGCCATCCTGCCAGGTTACATCGCATAGTGCCCCGGCTC 228

************************************************************

ASM-1 CGAGATGTCTTTGGGTGGGGGAACCTCACCTGCCCAATCTGCAAAGGTCTATTCACCGCC 300

ASM-5 CGAGATGTCTTTGGGTGGGGGAACCTCACCTGCCCAATCTGCAAAGGTCTATTCACCGCC 294

ASM-6 CGAGATGTCTTTGGGTGGGGGAACCTCACCTGCCCAATCTGCAAAGGTCTATTCACCGCC 294

ASM-7 CGAGATGTCTTTGGGTGGGGGAACCTCACCTGCCCAATCTGCAAAGGTCTATTCACCGCC 288

************************************************************

ASM-1 ATCAACCTCGGGCTGAAGAAGGAACCCAATGTGGCTCGCGTGGGCTCCGTGGCCATCAAG 360

ASM-5 ATCAACCTCGGGCTGAAGAAGGAACCCAATGTGGCTCGCGTGGGCTCCGTGGCCATCAAG 354

ASM-6 ATCAACCTCGGGCTGAAGAAGGAACCCAATGTGGCTCGCGTGGGCTCCGTGGCCATCAAG 354

ASM-7 ATCAACCTCGGGCTGAAGAAGGAACCCAATGTGGCTCGCGTGGGCTCCGTGGCCATCAAG 348

************************************************************

ASM-1 CTGTGCAATCTGCTGAAGATAGCACCACCTGCCGTGTGCCAATCCATTGTCCACCTCTTT 420

ASM-5 CTGTGCAATCTGCTGAAGATAGCACCACCTGCCGTGTGCCAATCCATTGTCCACCTCTTT 414

ASM-6 CTGTGCAATCTGCTGAAGATAGCACCACCTGCCGTGTGCCAATCCATTGTCCACCTCTTT 414

ASM-7 CTGTGCAATCTGCTGAAGATAGCACCACCTGCCGTGTGCCAATCCATTGTCCACCTCTTT 408

************************************************************

ASM-1 GAGGATGACATGGTGGAGGTGTGGAGACGCTCAGTGCTGAGCCCATCTGAGGCCTGTGGC 480

ASM-5 GAGGATGACATGGTGGAGGTGTGGAGACGCTCAGTGCTGAGCCCATCTGAGGCCTGTGGC 474

ASM-6 GAGGATGACATGGTGGAGGTGTGGAGACGCTCAGTGCTGAGCCCATCTGAGGCCTGTGGC 474

ASM-7 GAGGATGACATGGTGGAGGTGTGGAGACGCTCAGTGCTGAGCCCATCTGAGGCCTGTGGC 468

************************************************************

ASM-1 CTGCTCCTGGGCTCCACCTGTGGGCACTGGGACATTTTCTCATCTTGGAACATCTCTTTG 540

ASM-5 CTGCTCCTGGGCTCCACCTGTGGGCACTGGGACATTTTCTCATCTTGGAACATCTCTTTG 534

ASM-6 CTGCTCCTGGGCTCCACCTGTGGGCACTGGGACATTTTCTCATCTTGGAACATCTCTTTG 534

ASM-7 CTGCTCCTGGGCTCCACCTGTGGGCACTGGGACATTTTCTCATCTTGGAACATCTCTTTG 528

************************************************************

ASM-1 CCTACTGTGCCGAAGCCGCCCCCCAAACCCCCTAGCCCCCCAGCCCCAGGTGCCCCTGTC 600

ASM-5 CCTACTGTGCCGAAGCCGCCCCCCAAACCCCCTAGCCCCCCAGCCCCAGGTGCCCCTGTC 594

ASM-6 CCTACTGTGCCGAAGCCGCCCCCCAAACCCCCTAGCCCCCCAGCCCCAGGTGCCCCTGTC 594

ASM-7 CCTACTGTGCCGAAGCCGCCCCCCAAACCCCCTAGCCCCCCAGCCCCAGGTGCCCCTGTC 588

************************************************************

ASM-1 AGCCGCATCCTCTTCCTCACTGACCTGCACTGGGATCATGACTACCTGGAGGGCACGGAC 660

ASM-5 AGCCGCATCCTCTTCCTCACTGACCTGCACTGGGATCATGACTACCTGGAGGGCACGGAC 654

ASM-6 AGCCGCATCCTCTTCCTCACTGACCTGCACTGGGATCATGACTACCTGGAGGGCACGGAC 654

ASM-7 AGCCGCATCCTCTTCCTCACTGACCTGCACTGGGATCATGACTACCTGGAGGGCACGGAC 648

************************************************************

ASM-1 CCTGACTGTGCAGACCCACTGTGCTGCCGCCGGGGTTCTGGCCTGCCGCCCGCATCCCGG 720

ASM-5 CCTGACTGTGCAGACCCACTGTGCTGCCGCCGGGGTTCTGGCCTGCCGCCCGCATCCCGG 714

ASM-6 CCTGACTGTGCAGACCCACTGTGCTGCCGCCGGGGTTCTGGCCTGCCGCCCGCATCCCGG 714

ASM-7 CCTGACTGTGCAGACCCACTGTGCTGCCGCCGGGGTTCTGGCCTGCCGCCCGCATCCCGG 708

************************************************************

ASM-1 CCAGGTGCCGGATACTGGGGCGAATACAGCAAGTGTGACCTGCCCCTGAGGACCCTGGAG 780

ASM-5 CCAGGTGCCGGATACTGGGGCGAATACAGCAAGTGTGACCTGCCCCTGAGGACCCTGGAG 774

ASM-6 CCAGGTGCCGGATACTGGGGCGAATACAGCAAGTGTGACCTGCCCCTGAGGACCCTGGAG 774

ASM-7 CCAGGTGCCGGATACTGGGGCGAATACAGCAAGTGTGACCTGCCCCTGAGGACCCTGGAG 768

************************************************************

ASM-1 AGCCTGTTGAGTGGGCTGGGCCCAGCCGGCCCTTTTGATATGGTGTACTGGACAGGAGAC 840

ASM-5 AGCCTGTTGAGTGGGCTGGGCCCAGCCGGCCCTTTTGATATGGTGTACTGGACAGGAGAC 834

ASM-6 AGCCTGTTGAGTGGGCTGGGCCCAGCCGGCCCTTTTGATATGGTGTACTGGACAGGAGAC 834

ASM-7 AGCCTGTTGAGTGGGCTGGGCCCAGCCGGCCCTTTTGATATGGTGTACTGGACAGGAGAC 828

************************************************************

ASM-1 ATCCCCGCACATGATGTCTGGCACCAGACTCGTCAGGACCAACTGCGGGCCCTGACCACC 900

ASM-5 ATCCCCGCACATGATGTCTGGCACCAGACTCGTCAGGACCAACTGCGGGCCCTGACCACC 894

ASM-6 ATCCCCGCACATGATGTCTGGCACCAGACTCGTCAGGACCAACTGCGGGCCCTGACCACC 894

ASM-7 ATCCCCGCACATGATGTCTGGCACCAGACTCGTCAGGACCAACTGCGGGCCCTGACCACC 888

************************************************************

ASM-1 GTCACAGCACTTGTGAGGAAGTTCCTGGGGCCAGTGCCAGTGTACCCTGCTGTGGGTAAC 960

ASM-5 GTCACAGCACTTGTGAGGAAGTTCCTGGGGCCAGTGCCAGTGTACCCTGCTGTGGGTAAC 954

ASM-6 GTCACAGCACTTGTGAGGAAGTTCCTGGGGCCAGTGCCAGTGTACCCTGCTGTGGGTAAC 954

ASM-7 GTCACAGCACTTGTGAGGAAGTTCCTGGGGCCAGTGCCAGTGTACCCTGCTGTGGGTAAC 948

************************************************************

ASM-1 CATGAAAGCACACCTGTCAATAGCTTCCCTCCCCCCTTCATTGAGGGCAACCACTCCTCC 1020

ASM-5 CATGAAAGCACACCTGTCAATAGCTTCCCTCCCCCCTTCATTGAGGGCAACCACTCCTCC 1014

ASM-6 CATGAAAGCACACCTGTCAATAGCTTCCCTCCCCCCTTCATTGAGGGCAACCACTCCTCC 1014

ASM-7 CATGAAAGCACACCTGTCAATAGCTTCCCTCCCCCCTTCATTGAGGGCAACCACTCCTCC 1008

************************************************************

ASM-1 CGCTGGCTCTATGAAGCGATGGCCAAGGCTTGGGAGCCCTGGCTGCCTGCCGAAGCCCTG 1080

ASM-5 CGCTGGCTCTATGAAGCGATGGCCAAGGCTTGGGAGCCCTGGCTGCCTGCCGAAGCCCTG 1074

ASM-6 CGCTGGCTCTATGAAGCGATGGCCAAGGCTTGGGAGCCCTGGCTGCCTGCCGAAGCCCTG 1074

ASM-7 CGCTGGCTCTATGAAGCGATGGCCAAGGCTTGGGAGCCCTGGCTGCCTGCCGAAGCCCTG 1068

************************************************************

ASM-1 CGCACCCTCAG----------------------------------------AATTGGGGG 1100

ASM-5 CGCACCCTCAG------------------------------------------------- 1085

ASM-6 CGCACCCTCAG----------------------------------------AATTGGGGG 1094

ASM-7 CGCACCCTCAGGTACTTATCGTCCGTGGAAACCCAGGAAGGGAAAAGAAAGAATTGGGGG 1128

***********

ASM-1 GTTCTATGCTCTTTCCCCATACCCCGGTCTCCGCCTCATCTCTCTCAATATGAATTTTTG 1160

ASM-5 ------------------------------------------------------------

ASM-6 GTTCTATGCTCTTTCCCCATACCCCGGTCTCCGCCTCATCTCTCTCAATATGAATTTTTG 1154

ASM-7 GTTCTATGCTCTTTCCCCATACCCCGGTCTCCGCCTCATCTCTCTCAATATGAATTTTTG 1188

ASM-1 TTCCCGTGAGAACTTCTGGCTCTTGATCAACTCCACGGATCCCGCAGGACAGCTCCAGTG 1220

ASM-5 TTCCCGTGAGAACTTCTGGCTCTTGATCAACTCCACGGATCCCGCAGGACAGCTCCAGTG 1145

ASM-6 TTCCCGTGAGAACTTCTGGCTCTTGATCAACTCCACGGATCCCGCAGGACAGCTCCAGTG 1214

ASM-7 TTCCCG**TGA**GAACTTCTGGCTCTTGATCAACTCCACGGATCCCGCAGGACAGCTCCAGTG 1248

************************************************************

ASM-1 GCTGGTGGGGGAGCTTCAGGCTGCTGAGGATCGAGGAGACAAAGTGCATATAATTGGCCA 1280

ASM-5 GCTGGTGGGGGAGCTTCAGGCTGCTGAGGATCGAGGAGACAAAGTGCATATAATTGGCCA 1205

ASM-6 GCTGGTGGGGGAGCTTCAGGCTGCTGAGGATCGAGGAGACAAAGTGCATATAATTGGCCA 1274

ASM-7 GCTAGTGGGGGAGCTTCAGGCTGCTGAGGATCGAGGAGACAAAGTGCATATAATTGGCCA 1308

*** ********************************************************

ASM-1 CATTCCCCCAGGGCACTGTCTGAAGAGCTGGAGCTGGAATTATTACCGAATTGTAGCCAG 1340

ASM-5 CATTCCCCCAGGGCACTGTCTGAAGAGCTGGAGCTGGAATTATTACCGAATTGTAGCCAG 1265

ASM-6 CATTCCCCCAGGGCACTGTCTGAAGAGCTGGAGCTGGAATTATTACCGAATTGTAGCCAG 1334

ASM-7 CATTCCCCCAGGGCACTGTCTGAAGAGCTGGAGCTGGAATTATTACCGAATTGTAGCCAG 1368

************************************************************

ASM-1 GTATGAGAACACCCTGGCTGCTCAGTTCTTTGGCCACACTCATGTGGATGAATTTGAGGT 1400

ASM-5 GTATGAGAACACCCTGGCTGCTCAGTTCTTTGGCCACACTCATGTGGATGAATTTGAGGT 1325

ASM-6 GTATGAGAACACCCTGGCTGCTCAGTTCTTTGGCCACACTCATGTGGATGAATTTGAGGT 1394

ASM-7 GTATGAGAACACCCTGGCTGCTCAGTTCTTTGGCCACACTCATGTGGATGAATTTGAGGT 1428

************************************************************

ASM-1 CTTCTATGATGAAGAGACTCTGAGCCGGCCGCTGGCTGTAGCCTTCCTGGCACCCAGTGC 1460

ASM-5 CTTCTATGATGAAGAGACTCTGAGCCGGCCGCTGGCTGTAGCCTTCCTGGCACCCAGTGC 1385

ASM-6 CTTCTATGATGAAGAGACTCTGAGCCGGCCGCTGGCTGTAGCCTTCCTGGCACCCAGTGC 1454

ASM-7 CTTCTATGATGAAGAGACTCTGAGCCGGCCGCTGGCTGTAGCCTTCCTGGCACCCAGTGC 1488

************************************************************

ASM-1 AACTACCTACATCGGCCTTAATCCTG--------------------GTTACCGTGTGTAC 1500

ASM-5 AACTACCTACATCGGCCTTAATCCTG--------------------GTTACCGTGTGTAC 1425

ASM-6 AACTACCTACATCGGCCTTAATCCTGTCAGCCCCACATCCTTGCAGGTTACCGTGTGTAC 1514

ASM-7 AACTACCTACATCGGCCTTAATCCTG--------------------GTTACCGTGTGTAC 1528

************************** **************

ASM-1 CAAATAGATGGAAACTACTCCGGGAGCTCTCACGTGGTCCTGGACCATGAGACCTACATC 1560

ASM-5 CAAA**TAG**ATGGAAACTACTCCAGGAGCTCTCACGTGGTCCTGGACCATGAGACCTACATC 1485

ASM-6 CAAATAGATGGAAACTACTCCAGGAGCTCTCACGTGGTCCTGGACCATGAGACCTACATC 1574

ASM-7 CAAATAGATGGAAACTACTCCGGGAGCTCTCACGTGGTCCTGGACCATGAGACCTACATC 1588

********************* **************************************

ASM-1 CTGAATCTGACCCAGGCAAACATACCGGGAGCCATACCGCACTGGCAGCTTCTCTACAGG 1620

ASM-5 CTGAATCTGACCCAGGCAAACATACCGGGAGCCATACCGCACTGGCAGCTTCTCTACAGG 1545

ASM-6 CTGAATCTGACCCAGGCAAACATACCGGGAGCCATACCGCACTGGCAGCTTCTCTACAGG 1634

ASM-7 CTGAATCTGACCCAGGCAAACATACCGGGAGCCATACCGCACTGGCAGCTTCTCTACAGG 1648

************************************************************

ASM-1 GCTCGAGAAACCTATGGGCTGCCCAACACACTGCCTACCGCCTGGCACAACCTGGTATAT 1680

ASM-5 GCTCGAGAAACCTATGGGCTGCCCAACACACTGCCTACCGCCTGGCACAACCTGGTATAT 1605

ASM-6 GCTCGAGAAACCTATGGGCTGCCCAACACACTGCCTACCGCCTGGCACAACCTGGTATAT 1694

ASM-7 GCTCGAGAAACCTATGGGCTGCCCAACACACTGCCTACCGCCTGGCACAACCTGGTATAT 1708

************************************************************

ASM-1 CGCATGCGGGGCGACATGCAACTTTTCCAGACCTTCTGGTTTCTCTACCATAAGGGCCAC 1740

ASM-5 CGCATGCGGGGCGACATGCAACTTTTCCAGACCTTCTGGTTTCTCTACCATAAGGGCCAC 1665

ASM-6 CGCATGCGGGGCGACATGCAACTTTTCCAGACCTTCTGGTTTCTCTACCATAAGGGCCAC 1754

ASM-7 CGCATGCGGGGCGACATGCAACTTTTCCAGACCTTCTGGTTTCTCTACCATAAGGGCCAC 1768

************************************************************

ASM-1 CCACCCTCGGAGCCCTGTGGCACGCCCTGCCGTCTGGCTACTCTTTGTGCCCAGCTCTCT 1800

ASM-5 CCACCCTCGGAGCCCTGTGGCACGCCCTGCCGTCTGGCTACTCTTTGTGCCCAGCTCTCT 1725

ASM-6 CCACCCTCGGAGCCCTGTGGCACGCCCTGCCGTCTGGCTACTCTTTGTGCCCAGCTCTCT 1814

ASM-7 CCACCCTCGGAGCCCTGTGGCACGCCCTGCCGTCTGGCTACTCTTTGTGCCCAGCTCTCT 1828

************************************************************

ASM-1 GCCCGTGCTGACAGCCCTGCTCTGTGCCGCCACCTGATGCCAGATGGGAGCCTCCCAGAG 1860

ASM-5 GCCCGTGCTGACAGCCCTGCTCTGTGCCGCCACCTGATGCCAGATGGGAGCCTCCCAGAG 1785

ASM-6 GCCCGTGCTGACAGCCCTGCTCTGTGCCGCCACCTGATGCCAGATGGGAGCCTCCCAGAG 1874

ASM-7 GCCCGTGCTGACAGCCCTGCTCTGTGCCGCCACCTGATGCCAGATGGGAGCCTCCCAGAG 1888

************************************************************

ASM-1 GCCCAGAGCCTGTGGCCAAGGCCACTGTTTTGC**TAG** 1896

ASM-5 GCCCAGAGCCTGTGGCCAAGGCCACTGTTTTGC**TAG** 1821

ASM-6 GCCCAGAGCCTGTGGCCAAGGCCACTGTTTTGCTAG 1910

ASM-7 GCCCAGAGCCTGTGGCCAAGGCCACTGTTTTGCTAG 1924

************************************
