# Supplementary material for: Functional Implications of Novel Human Acid Sphingomyelinase Splice Variants
Source: PLoS One. 2012 Apr 27;7(4):e35467. doi: 10.1371/journal.pone.0035467 (PMC3338701; doi:10.1371/journal.pone.0035467)
Supplement: Figure S2 — The putative protein sequence of each novel isoform displays specific features. In comparison with the full-length ASM-1 protein, which consists of 631 amino acids and has a theoretical molecular weight of 70 kDa, ASM-5 is 606 amino acids long and has a theoretical molecular weight of 67 kDa. It carries a disrupted catalytic domain due to the loss of 23 amino acids. ASM-6 constitutes a 506 amino acid protein with a theoretical molecular weight of 56 kDa. It has an intact catalytic domain but lacks the C-terminal domain. Instead, it carries a unique C-terminal peptide composed of 13 amino acids, VSPTSLQVTVCTK. ASM-7 consists of 398 amino acids and has a theoretical molecular weight of 45 kDa. Like ASM-6, it is a smaller protein because of a truncated open reading frame. ASM-7 has a partial catalytic domain and entirely lacks the C-terminal domain of ASM-1. At its C-terminus, it carries a unique peptide of 38 amino acids (YLSSVETQEGKRKNWGVLCSFPIPRSPPHLSQYEFLFP). Specific protein sequences are indicated in grey. The protein domains are coloured differently: yellow, signal peptide (aa 1–48); light blue, saposin-B-domain (aa 91–167); purple, proline-rich-domain (aa 168–200); red, catalytic domain (aa 201–463); green, C-terminal domain (aa 464–631). Mannose-6-phosphate sites (Asn 88, 177, 337, 397, 505, 522) are indicated in light green. The alignment was performed using ClustalW. (DOC) [file pone.0035467.s002.doc]

ASM-1 MPRYGASLRQSCPRSGREQGQDGTAGAPGLLWMGLVLALALALALALALSDSRVLWAPAE 60

ASM-5 MPRYGASLRQSCPRSGREQGQDGTAGAPGLLWMGLVLALALALALA--LSDSRVLWAPAE 58

ASM-6 MPRYGASLRQSCPRSGREQGQDGTAGAPGLLWMGLVLALALALALA--LSDSRVLWAPAE 58

ASM-7 MPRYGASLRQSCPRSGREQGQDGTAGAPGLLWMG--LALALALALA--LSDSRVLWAPAE 56

********************************** ********** ************

ASM-1 AHPLSPQGHPARLHRIVPRLRDVFGWGNLTCPICKGLFTAINLGLKKEPNVARVGSVAIK 120

ASM-5 AHPLSPQGHPARLHRIVPRLRDVFGWGNLTCPICKGLFTAINLGLKKEPNVARVGSVAIK 118

ASM-6 AHPLSPQGHPARLHRIVPRLRDVFGWGNLTCPICKGLFTAINLGLKKEPNVARVGSVAIK 118

ASM-7 AHPLSPQGHPARLHRIVPRLRDVFGWGNLTCPICKGLFTAINLGLKKEPNVARVGSVAIK 116

************************************************************

ASM-1 LCNLLKIAPPAVCQSIVHLFEDDMVEVWRRSVLSPSEACGLLLGSTCGHWDIFSSWNISL 180

ASM-5 LCNLLKIAPPAVCQSIVHLFEDDMVEVWRRSVLSPSEACGLLLGSTCGHWDIFSSWNISL 178

ASM-6 LCNLLKIAPPAVCQSIVHLFEDDMVEVWRRSVLSPSEACGLLLGSTCGHWDIFSSWNISL 178

ASM-7 LCNLLKIAPPAVCQSIVHLFEDDMVEVWRRSVLSPSEACGLLLGSTCGHWDIFSSWNISL 176

************************************************************

ASM-1 PTVPKPPPKPPSPPAPGAPVSRILFLTDLHWDHDYLEGTDPDCADPLCCRRGSGLPPASR 240

ASM-5 PTVPKPPPKPPSPPAPGAPVSRILFLTDLHWDHDYLEGTDPDCADPLCCRRGSGLPPASR 238

ASM-6 PTVPKPPPKPPSPPAPGAPVSRILFLTDLHWDHDYLEGTDPDCADPLCCRRGSGLPPASR 238

ASM-7 PTVPKPPPKPPSPPAPGAPVSRILFLTDLHWDHDYLEGTDPDCADPLCCRRGSGLPPASR 236

************************************************************

ASM-1 PGAGYWGEYSKCDLPLRTLESLLSGLGPAGPFDMVYWTGDIPAHDVWHQTRQDQLRALTT 300

ASM-5 PGAGYWGEYSKCDLPLRTLESLLSGLGPAGPFDMVYWTGDIPAHDVWHQTRQDQLRALTT 298

ASM-6 PGAGYWGEYSKCDLPLRTLESLLSGLGPAGPFDMVYWTGDIPAHDVWHQTRQDQLRALTT 298

ASM-7 PGAGYWGEYSKCDLPLRTLESLLSGLGPAGPFDMVYWTGDIPAHDVWHQTRQDQLRALTT 296

************************************************************

ASM-1 VTALVRKFLGPVPVYPAVGNHESTPVNSFPPPFIEGNHSSRWLYEAMAKAWEPWLPAEAL 360

ASM-5 VTALVRKFLGPVPVYPAVGNHESTPVNSFPPPFIEGNHSSRWLYEAMAKAWEPWLPAEAL 358

ASM-6 VTALVRKFLGPVPVYPAVGNHESTPVNSFPPPFIEGNHSSRWLYEAMAKAWEPWLPAEAL 358

ASM-7 VTALVRKFLGPVPVYPAVGNHESTPVNSFPPPFIEGNHSSRWLYEAMAKAWEPWLPAEAL 356

************************************************************

ASM-1 RTLRIGGFYALSPYPGLRLISLNMNFCSRENFWLLINSTDPAGQLQWLVGELQAAEDRGD 420

ASM-5 RTL-----------------------SSRENFWLLINSTDPAGQLQWLVGELQAAEDRGD 395

ASM-6 RTLRIGGFYALSPYPGLRLISLNMNFCSRENFWLLINSTDPAGQLQWLVGELQAAEDRGD 418

ASM-7 RTLRYLSSVETQEGKRKNWGVLCSFPIPRSPPHLSQYEFLFP------------------ 398

***

ASM-1 KVHIIGHIPPGHCLKSWSWNYYRIVARYENTLAAQFFGHTHVDEFEVFYDEETLSRPLAV 480

ASM-5 KVHIIGHIPPGHCLKSWSWNYYRIVARYENTLAAQFFGHTHVDEFEVFYDEETLSRPLAV 455

ASM-6 KVHIIGHIPPGHCLKSWSWNYYRIVARYENTLAAQFFGHTHVDEFEVFYDEETLSRPLAV 478

ASM-7 ------------------------------------------------------------

ASM-1 AFLAPSATTYIGLNPGYRVYQIDGNYSGSSHVVLDHETYILNLTQANIPGAIPHWQLLYR 540

ASM-5 AFLAPSATTYIGLNPGYRVYQIDGNYSRSSHVVLDHETYILNLTQANIPGAIPHWQLLYR 515

ASM-6 AFLAPSATTYIGLNPVSPTSLQVTVCTK-------------------------------- 506

ASM-7 ------------------------------------------------------------

ASM-1 ARETYGLPNTLPTAWHNLVYRMRGDMQLFQTFWFLYHKGHPPSEPCGTPCRLATLCAQLS 600

ASM-5 ARETYGLPNTLPTAWHNLVYRMRGDMQLFQTFWFLYHKGHPPSEPCGTPCRLATLCAQLS 575

ASM-6 --------- --------------------------------------------------

ASM-7 ------------------------------------------------------------

ASM-1 ARADSPALCRHLMPDGSLPEAQSLWPRPLFC 631

ASM-5 ARADSPALCRHLMPDGSLPEAQSLWPRPLFC 606

ASM-6 -------------------------------

ASM-7 -------------------------------
