# Supplementary figures and images for: Functional Implications of Novel Human Acid Sphingomyelinase Splice Variants
Source: PLoS One. 2012 Apr 27;7(4):e35467. doi: 10.1371/journal.pone.0035467 (PMC3338701; doi:10.1371/journal.pone.0035467)

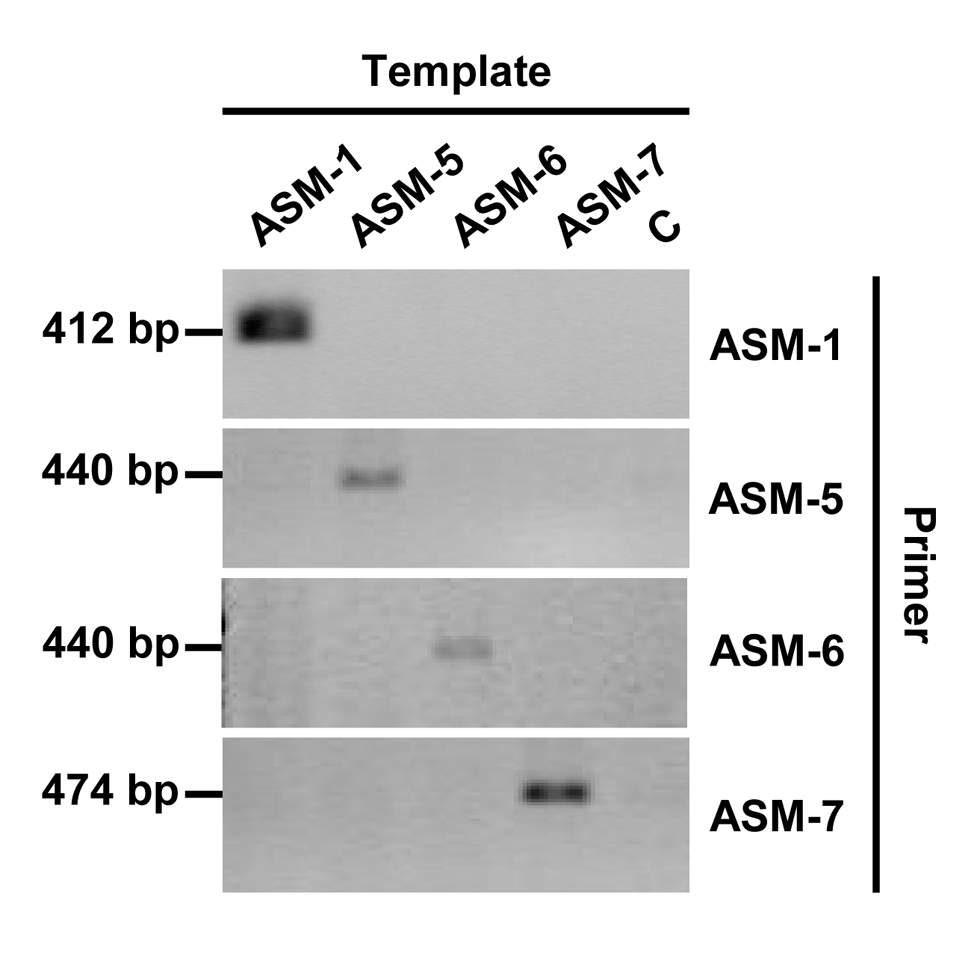

Supplement: Figure S3 — Target-specificity of primer pairs designed for amplification of ASM transcripts. Amplification of ASM-1 and each novel ASM transcript using the respective primer pair resulted in a specific PCR-product of the expected size separated on a 1% agarose gel. Cloned transcripts were used as templates; water served as a negative control (C). Amplimers were verified by sequence analysis. (TIF) [file pone.0035467.s003.tif]
