# Supplementary material for: Functional Implications of Novel Human Acid Sphingomyelinase Splice Variants
Source: PLoS One. 2012 Apr 27;7(4):e35467. doi: 10.1371/journal.pone.0035467 (PMC3338701; doi:10.1371/journal.pone.0035467)
Supplement: Table S1 — MALDI-TOF MS detection of sphingomyelin and ceramide species for semi-quantitative analysis. (DOC) [file pone.0035467.s004.doc]

**Table S1**. MALDI-TOF MS detection of sphingomyelin and ceramide species for semi-quantitative analysis.

| **Characteristic ions (m/z)** | **Molecular species** | **Designation** | **Adduct** |
| --- | --- | --- | --- |
|  |  |  |  |
| **Sphingomyelin** |  |  |  |
| 647 | (d18:1/12:0) | N-(dodecanoyl)-sphing-4-enine-1-phosphocholine | H^+^ |
| 685 | (d18:1/12:0) | N-(dodecanoyl)-sphing-4-enine-1-phosphocholine | K^+^ |
| 703 | (d18:1/16:0) | N-(hexadecanoyl)-sphing-4-enine-1-phosphocholine | H^+^ |
| 725 | (d18:1/16:0) | N-(hexadecanoyl)-sphing-4-enine-1-phosphocholine | Na^+^ |
| 731 | (d18:1/18:0) | N-(octadecanoyl)-sphing-4-enine-1-phosphocholine | H^+^ |
| 753 | (d18:1/18:0) | N-(octadecanoyl)-sphing-4-enine-1-phosphocholine | Na^+^ |
| 745 | (d18:1/19:0) | N-(nonadecanoyl)-sphing-4-enine-1-phosphocholine | H^+^ |
| 767 | (d18:1/19:0) | N-(nonadecanoyl)-sphing-4-enine-1-phosphocholine | Na^+^ |
| 759 | (d18:1/20:0) | N-(eicosanoyl)-sphing-4-enine-1-phosphocholine | H^+^ |
| 781 | (d18:1/20:0) | N-(eicosanoyl)-sphing-4-enine-1-phosphocholine | Na^+^ |
|  |  |  |  |
| **Ceramide** |  |  |  |
| 481 | (d18:1/12:0) | N-(dodecanoyl)-sphing-4-enine | H^+^ |
| 578 | (d18:1/12:0) | N-(dodecanoyl)-sphing-4-enine-1-phosphate, NH_4_ salt | H^+^ |
| 537 | (d18:1/16:0) | N-(hexadecanoyl)-sping-4-enine | H^+^ |
| 617 | (d18:1/16:0) | N-(hexadecanoyl)-sping-4-enine-1-phosphate | H^+^ |
| 565 | (d18:1/18:0) | N-(octadecanoyl)-sping-4-enine | H^+^ |
| 593 | (d18:1/20:0) | N-(eicosanoyl)-sping-4-enine | H^+^ |

Characteristic ions detected by MALDI-TOF MS.
