# Supplementary material for: Functional Implications of Novel Human Acid Sphingomyelinase Splice Variants
Source: PLoS One. 2012 Apr 27;7(4):e35467. doi: 10.1371/journal.pone.0035467 (PMC3338701; doi:10.1371/journal.pone.0035467)
Supplement: Table S2 — RT-qPCR parameters according to the MIQE précis checklist. (DOC) [file pone.0035467.s005.doc]

**Table S2. RT-qPCR parameters according to the MIQE précis checklist.**

| **Sample/Template** |  |
| --- | --- |
| Source | Human THP-1 / H4 cells |
| Method of preservation | Direct isolation |
| Storage time | Direct isolation |
| Handling | Direct isolation |
| Extraction method | High Pure RNA Isolation Kit (Roche, Mannheim, Germany) / Qiazol (Qiagen, Hilden, Germany) |
| RNA: DNA-free | Intron-spanning primers |
| Concentration | Nanodrop (Peqlab, Erlangen, Germany) |
| RNA: integrity | Experion (Bio-Rad, Munich, Germany) |
| **Assay validation** |  |
| Accession number | See table 2 |
| Amplicon details | See table 2 |
| Primer sequence | See table 2 |
| *In silico* | Primer BLAST |
| Empirical | Primer concentration, annealing temperature, target specificity |
| Priming conditions | Combination oligo-dT/random |
| PCR efficiency | Dilution curves |
| **RT/PCR** |  |
| Protocols | See materials and methods |
| Reagents | See materials and methods |
| No template control | Melt curves |
| Positive control | Inter-run calibrator |
| **Data analysis** |  |
| Specialist software | Light cycler 480 (Roche, Mannheim, Germany) |
| Normalisation | Inter-run calibrator |

Checklist for qPCR analyses derived from Bustin *et al.* [48].
